# Risk of Atrial Fibrillation Following Left Bundle Branch Area Pacing versus Right Ventricular Pacing and Biventricular Pacing: A Systematic Review and Meta-Analysis

**DOI:** 10.31083/j.rcm2408220

**Published:** 2023-08-01

**Authors:** Bing Liu, Wenlong Dai, Yake Lou, Yulin Li, Yongquan Wu, Jie Du

**Affiliations:** ^1^Department of Cardiology, Beijing Anzhen Hospital, Capital Medical University, 100029 Beijing, China; ^2^Department of Cardiology, The Second Affiliated Hospital of Chongqing Medical University, 400010 Chongqing, China; ^3^Beijing Institute of Heart, Lung and Blood Vessel Diseases, Beijing Anzhen Hospital, Capital Medical University, 100029 Beijing, China

**Keywords:** atrial fibrillation, left bundle branch area pacing, left bundle branch pacing, biventricular pacing, right ventricular pacing, meta-analysis, systematic review

## Abstract

**Background:**

Left bundle branch pacing (LBBP) is a relatively novel physiological pacing strategy with better electrocardiogram characteristics and pacing parameters than other pacing strategies. At present, no meta-analysis or systematic review has examined the risk of atrial fibrillation (AF) after LBBP compared to other pacing strategies.

**Methods:**

We searched the PubMed, Embase, and Cochrane Library databases from inception through September 18, 2022 to identify relevant studies reporting AF incidence rates after LBBP. The incidence of AF following LBBP and that associated with other pacing strategies were extracted and summarized for the meta-analysis. We used odds ratios (ORs) and 95% confidence intervals (CIs) as summary estimates.

**Results:**

Five studies with 1144 participants were included. The pooled rate of AF was 3.7% (95% CI, 0.8%–8.0%) in the LBBP group and 15.5% (95% CI: 9.6%–22.4%) in the other pacing strategies (right ventricular pacing [RVP] and biventricular pacing [BVP]). Compared with other pacing strategies, LBBP was associated with a lower AF risk (OR, 0.33; 95% CI: 0.22–0.51, $I^{2}$ = 0.0%; *p* = 0.485). Similar results were observed for LBBP when compared with RVP (OR: 0.33, 95% CI: 0.22–0.51, $I^{2}$ = 0.0%, *p* = 0.641) and BVP (OR: 0.47, 95% CI: 0.01–15.22, $I^{2}$ = 60.4%, *p* = 0.112).

**Conclusions:**

Compared with BVP and RVP, LBBP was associated with a significantly lower risk of AF. However, further large-sample randomized controlled trials are needed to confirm that LBBP is superior to other pacing strategies in reducing AF risk.

## 1. Introduction

Right ventricular pacing (RVP) is recommended for patients with symptomatic bradyarrhythmia and cardiac conduction dysfunction [1]. Although RVP is a well-established pacing strategy in clinical practice, it has been demonstrated that chronic RVP may lead to electrical and mechanical dyssynchrony and is associated with an increased risk of atrial fibrillation (AF), heart failure (HF) hospitalization, left ventricular (LV) dysfunction, and increased mortality [2, 3, 4]. Cardiac resynchronization therapy (CRT), which improves mechanical dyssynchrony through the electrical activation of the heart in a coordinated manner, can overcome the limitations of RVP; it is mainly used to treat patients with HF and ventricular systolic dyssynchrony [5, 6]. While it has been established that CRT with biventricular pacing (BVP) is superior to RVP in patients with atrioventricular block and reduced left ventricular ejection fraction [7], patient response to BVP was variable and 30%–40% of patients did not experience any benefit from BVP, including patients with narrow QRS duration and those with right bundle branch block [8, 9, 10]. Subsequently, two physiological pacing strategies, His bundle pacing (HBP) and left bundle branch pacing (LBBP), have become effective alternatives to CRT. HBP, a feasible alternative to CRT, directly paces the His-Purkinje system to activate the ventricles and physiologically achieve synchronous contraction [11, 12]. However, its steeper and longer learning curve, higher pacing thresholds, and lower implantation success rates have limited its clinical application [13]. Therefore, as a novel pacing technology first reported by Huang *et al*. [14] in 2017, LBBP directly captures the left bundle branch through deep septal pacing and is gradually being widely used in clinical practice because of its low pacing threshold, lead stability, normal ventricular sensing, and correction of distal conduction system disease [15].

Several studies have demonstrated the effectiveness and safety of LBBP. Meta-analyses have demonstrated that compared with other pacing strategies, LBBP was associated with better performance in pacing parameters and improved clinical outcomes, such as a lower capture threshold and larger R-wave amplitude at implantation, shortened QRS duration, and greater improvement in LVEF [16, 17, 18, 19]. While most studies mainly focus on the electrocardiogram characteristics and pacing parameters of LBBP compared with those of other pacing strategies [20, 21, 22], only few studies report the risk of AF in LBBP; in addition, the sample sizes of these studies were relatively small [23, 24, 25, 26]. To the best of our knowledge, only one meta-analysis reported as a conference abstract showed that physiological pacing (including HBP and LBBP) was not associated with AF risk reduction compared with RVP (odds ratio (OR) 0.95, 95% CI 0.76–1.18) [27]. No meta-analysis has specifically evaluated the risk of AF in patients receiving LBBP compared with other pacing strategies.

A study investigating the predictors of AF in patients with pacemakers found that pacemaker detected AF in 51.8% of patients without AF history during a mean follow-up of 52 months [28]. LBBP-associated improvements in biventricular synchrony and atrial function can theoretically reduce the risk of AF. Therefore, this systematic review and meta-analysis aimed to compare AF risk associated with LBBP with that of other pacing strategies.

## 2. Methods

This systematic review and meta-analysis was conducted according to the Preferred Reporting Items for Systematic Reviews and Meta-Analyses (PRISMA) statement and registered in PROSPERO database (CRD42022367476) [29].

### 2.1 Search Strategy and Selection Criteria

PubMed, Embase, and Cochrane Library were systematically searched from their inception dates to September 18, 2022. For a more comprehensive literature search, the search strategy included the following keywords: ‘left bundle branch pacing’ and ‘left bundle branch area pacing’. Eligible studies were included based on the following criteria: (1) the occurrence of AF was reported in the LBBP group and (2) studies published in English with an available full text. To obtain additional literature, we included conference abstracts and letters in the meta-analysis if they reported AF incidence in the LBBP group. The exclusion criteria were: (1) reviews, meta-analyses, editorials, protocol of trials, and case reports and (2) the incidence of AF was reported but AF incidence rate was not distinguished in the group of LBBP or other pacing strategies.

### 2.2 Study Selection and Data Extraction

Two reviewers, BL and WLD, independently screened the literature, reviewed the titles and abstracts, and further scrutinize the full text to assess whether the studies could be enrolled in the meta-analysis. Any discrepancies were resolved by a discussion with a third reviewer (YKL). Two reviewers (YKL and YQW) independently extracted data using a standard data extraction form. The following data were obtained from the eligible studies: author name, publication year, country, study time, study design, participants, age, sex, comparison, duration of follow-up, and AF incidence rate. The quality and risk of bias of eligible studies were assessed by two independent reviewers using the Newcastle-Ottawa Scale (NOS) for observational studies and the Cochrane Collaboration’s tool for randomized controlled trials (RCTs) [30, 31]. A third reviewer resolved any disagreements. Studies with an NOS score >6 stars were considered to be of high quality.

### 2.3 Outcomes

The primary outcome was the risk of AF in LBBP compared with that in other pacing strategies. AF was defined according to the definitions of AF in each article. The second outcome was the incidence of AF in LBBP and other pacing strategies. Subgroup analyses were performed based on follow-up (short-term and long-term AF incidence rates), pacing strategies, and race (Asian and non-Asian). AF events with less than 1 year of follow-up were classified as short-term outcomes; otherwise, they were classified as long-term outcomes.

### 2.4 Statistical Analysis

Stata 15 software (StataCorp LP, College Station, TX, USA) was used for all statistical analyses. Odds ratios (OR) with 95% CIs were used as the summary estimates. Statistical heterogeneity among studies was assessed using the chi-squared and $I^{2}$ tests. $I^{2}$ ≤ 50% indicated small heterogeneity between studies, $I^{2}$ > 50% indicated moderate heterogeneity and $I^{2}$ > 75% indicated considerable heterogeneity. Data from each study were pooled using a random effects model. Funnel plots were used to analyze studies for the presence or absence of publication bias. In addition, we used the leave-one-out method to perform the sensitivity analysis. All *p* values were two-sided, with *p* < 0.05 considered significant.

## 3. Results

### 3.1 Study Selection and Characteristics

**Fig. 1.**
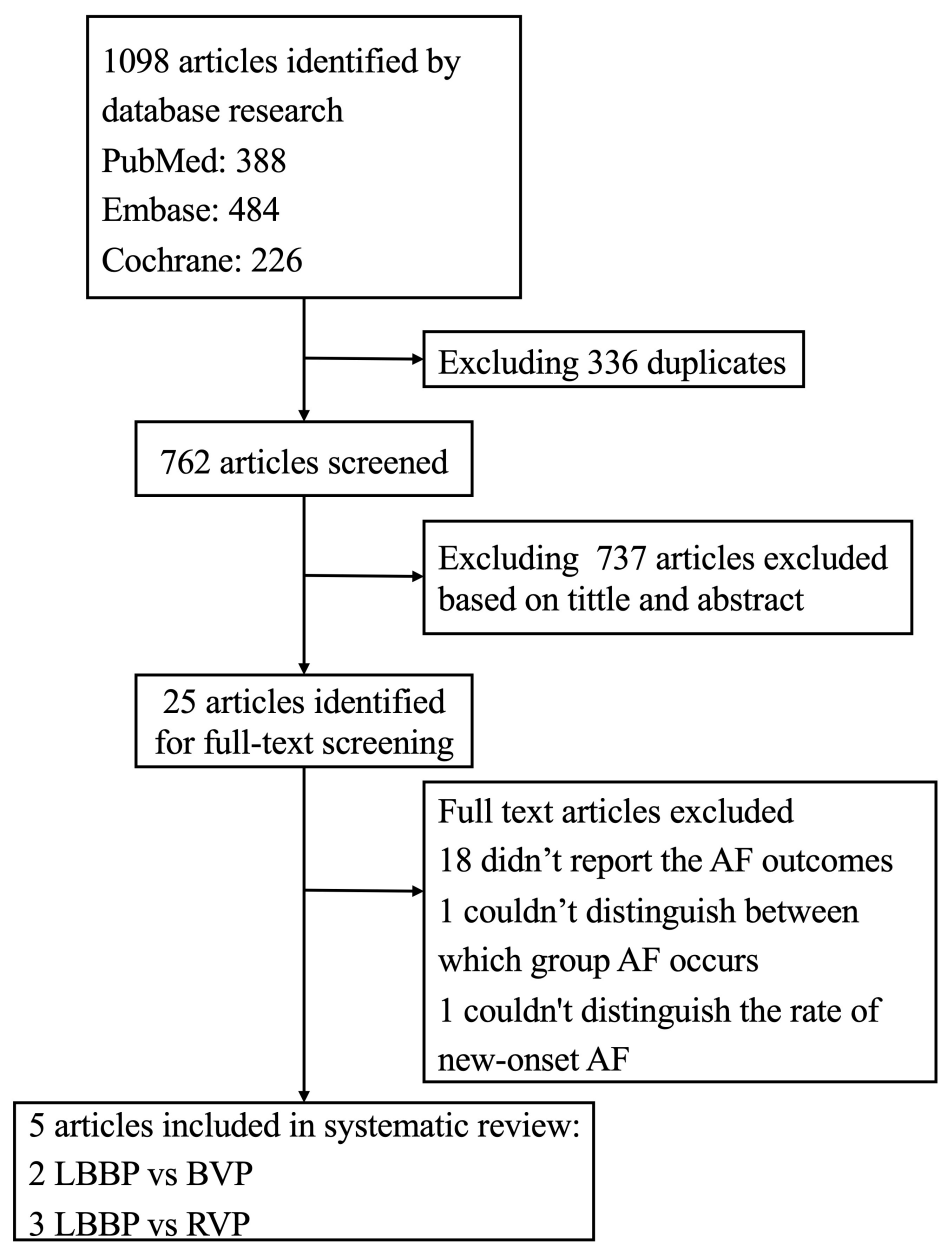
**Flowchart of study selection**. Abbreviations: AF, atrial fibrillation; LBBP, left bundle branch pacing; BVP, biventricular pacing; RVP, right ventricular pacing.

A total of 1098 articles were initially retrieved, of which 336 were duplicates. After screening titles and abstracts, 25 articles were identified for a full-text review. Based on the selection criteria, five articles were enrolled in the meta-analysis [23, 24, 25, 26, 32, 33]. The flow chart of the study selection process is manifested in Fig. 1. All the eligible studies were observational studies, among which two compared the occurrence of AF between LBBP and BVP [25, 26], and three reported AF incidence rate between LBBP and RVP [23, 24, 32]. There were four original articles [23, 25, 26, 32], and one letter [24]. The main characteristics of the five studies are summarized in Table 1 (Ref. [23, 24, 25, 26, 32]). Among three studies reported AF risk following LBBP and RVP, only one of them described the device programming [23]. For patients with sinus node dysfunction (SND) or intact atrioventricular (AV) conduction, automatic AV search algorithm was routinely turned on to avoid unnecessary ventricular pacing. AV delay was set based on intrinsic AV conduction to minimize conduction burden in patients with intermittent AV block. A default AV interval (180/150 ms quite often) was set for AV synchrony in patients with complete AV block.

**Table 1.** **Characteristics of the five eligible studies enrolled in the meta-analysis**.

Author (Year)	Country	Time	Study design	Patients	Age, years	Male, n (%)	Comparison (n)	Follow-up	AF rate	AF definitions
Chen (2022) [26]	China	January 2018 to September 2019	non-randomized, prospective, multi-centre, observational study	100 HF with reduced LVEF ≤35% and LBBB	LBBP:	54 (54.0)	LBBP (49) vs BVP (51)	6-month and	6-month:	Not reported
					67.1 ± 8.9			1-year	LBBP vs BVP:	
					BVP:				0 (0) vs 3 (5.89%)	
					64.4 ± 8.7				1-year:	
									LBBP vs BVP:	
									0 (0) vs 5 (9.80%)	
Hua (2022) [25]	China	February 2018 to May 2019	single-center, non-randomized, prospective observational study	41 HF with complete LBBB	LBBP:	30 (73.2)	LBBP (21) vs BVP (20)	24-month	LBBP vs BVP:	Not reported
					65.5 ± 6.9				1/16 (6.25%) vs 0/15 (0)	
					BVP:					
					67.5 ± 11.7					
Zhu (2022) [23]	China	June 2019 to November 2021	2-center, prospective observational cohort study	527 patients with bradycardia and indicated for dual-chamber pacemaker implantation, had no prior AF history (317 (60.2%) VP ≥20%)	65.3 ± 12.6	249 (47.3)	LBBP (257) vs RVP (270)	11.1 ± 7.5 months	LBBP vs RVP:	New-onset AF was defined as device-detected AF episodes lasting at least 30 s on intracardiac electrogram or surface 12-lead ECG.
							VP ≥20%:		7.4% vs 17.0%	
							LBBP (193): 75.1%			
							RVP (124): 45.9%			
Ravi (2022) [24]	America	April 2018 and October 2020	retrospective cohort study	410 patients with an age ≥18 years, seek for permanent pacemaker implantation with RVP and LBBP (281 (68.5%) VP ≥20%)	NA	NA	LBBP (173) vs RVP (237)	600 ± 278 days	AF ≥30 s:	New-onset AF episode ≥30 seconds detected on scheduled device follow-up performed in-person and remotely.
							VP ≥20%:		LBBP vs RVP:	
							LBBP (136): 78.6%		9 (5.2%) vs 43 (18.1%)	
							RVP (145): 61.2%			
Zhang (2021) [32]	China	January 2018 to December 2018	single-center, retrospective, observational study	66 AVB patients with indications for ventricular pacing (4 failed LBBP did not include in analysis)	65.5 ± 8.8	30 (66)	LBBP (29) vs RVP (37)	LBBP:	LBBP vs RVP:	New-onset AF was obtained via pacemaker program controller, defined as AF that lasted more than 30 s
								17.4 ± 3.4 months	4 (14.79%) vs 12 (32.43%)	
							Cum%VP:	RVP:		
							LBBP (95.47% ± 1.22%)	18.0 ± 3.3 months		
							RVP (94.86% ± 1.56%)			

HF, heart failure; LVEF, left ventricular ejection fraction; LBBB, left bundle branch block; LBBP, left bundle branch pacing; BVP, biventricular pacing; AF, atrial fibrillation; VP, ventricular pacing; Cum%VP, cumulative percentage of VP; RVP, right ventricular pacing; AVB, atrial ventricular block; NA, not available; ECG, Electrocardiograph.

All eligible studies were scored using the NOS quality assessment system. The five studies were of high quality, with NOS scores of >6 (Table 2, Ref. [23, 24, 25, 26, 32]).

**Table 2.** **The Newcastle-Ottawa Scale scores for the five included studies**.

Author (Year)	Representativeness of the exposed cohort	Selection of the non-exposed cohort	Ascertainment of exposure	Outcome of interest was not present at the start	Comparability	Assessment of outcome	Enough follow-up	Adequacy of follow-up	Total	Quality
Chen (2022) [26]	1	1	1	0	2	1	1	1	8	high
Hua (2022) [25]	1	1	1	0	2	1	1	0	7	high
Zhu (2022) [23]	1	1	1	1	2	1	1	1	9	high
Ravi (2022) [24]	1	1	1	1	2	1	1	1	9	high
Zhang (2021) [32]	1	1	1	1	2	1	1	1	9	high

### 3.2 AF Incidence Rate

**Fig. 2.**
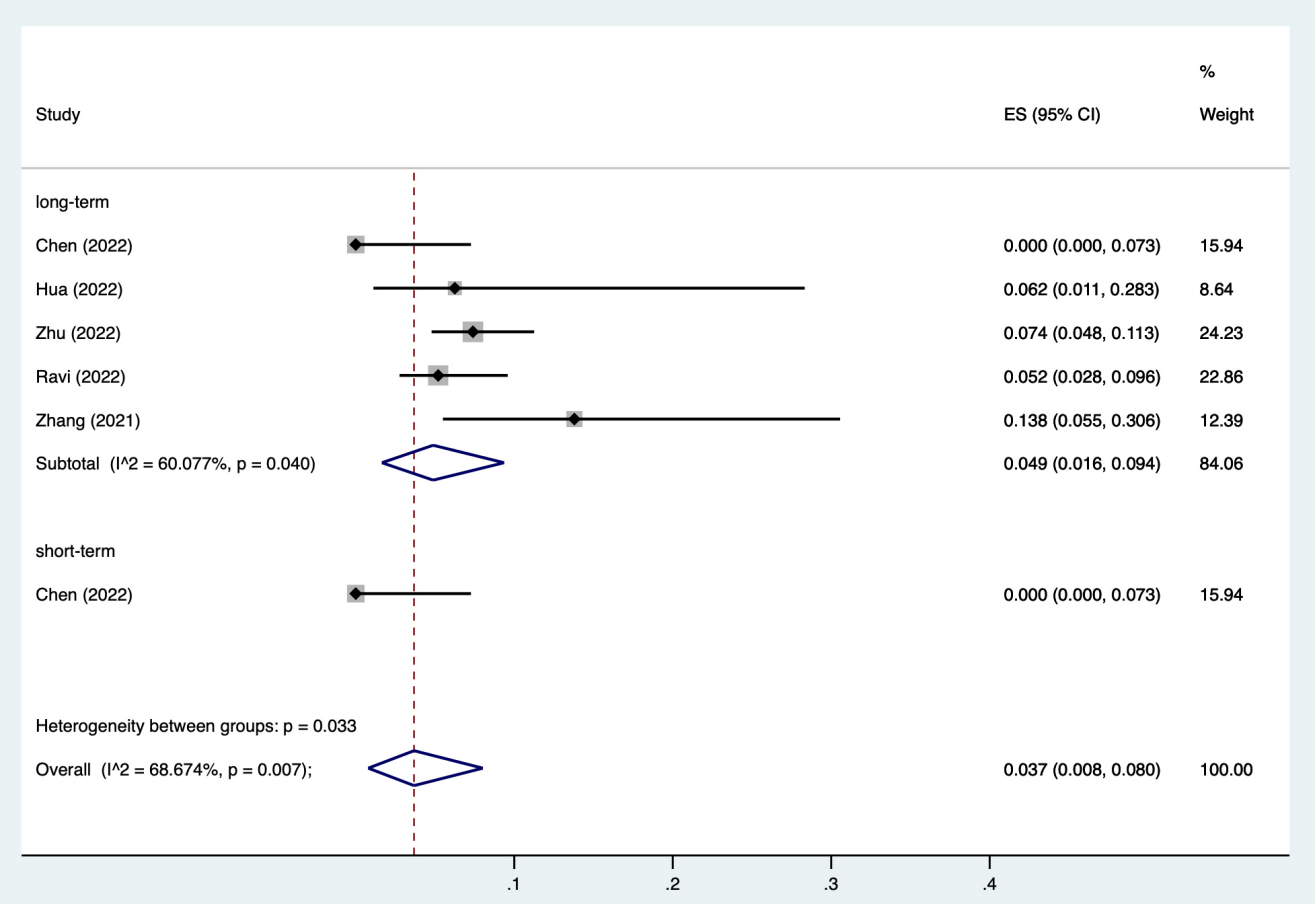
**Incidence rate of AF among patients receiving LBBP**. Abbreviations: AF, atrial fibrillation; LBBP, left bundle branch pacing; ES, effect size; CI, confidence interval.

All studies reported AF incidence rate in the LBBP group, among which one reported long-term and short-term AF incidence rates [26]. The pooled rate of AF was 3.7% (95% CI, 0.8%–8.0%), with a high heterogeneity $I^{2}$ of 68.7% (*p* = 0.007). After stratification of short-term and long-term outcomes, the results showed that the long-term AF incidence rate in the LBBP group was 4.9% (95% CI, 1.6%–9.4%) and short-term AF incidence rate was 0% (95% CI, 0%–7.3%) (Fig. 2).

**Fig. 3.**
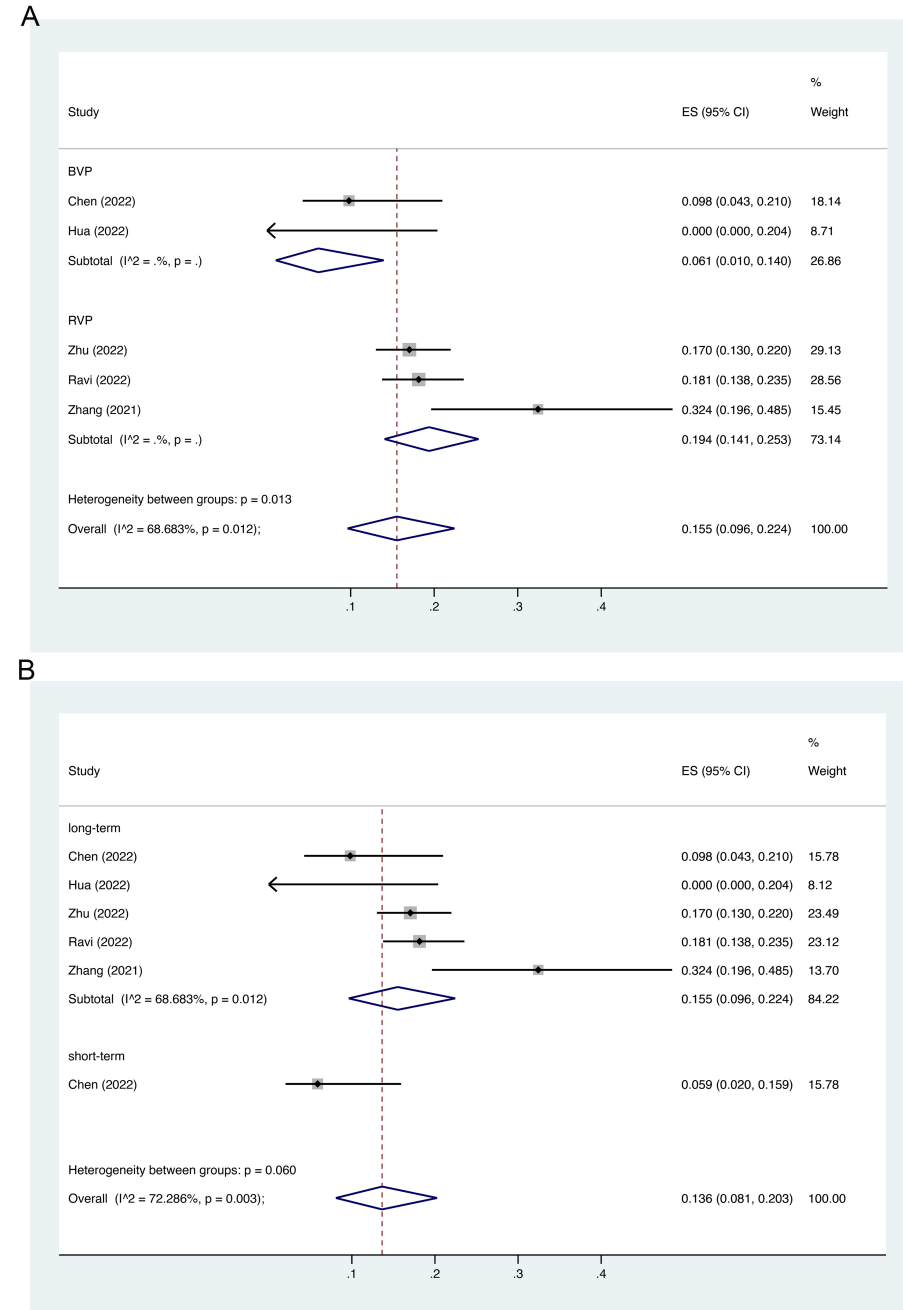
**Incidence rate of AF stratified by pacing strategies and follow-up time**. (A) AF incidence rate in BVP and RVP; (B) AF long-term incidence rate and short-term incidence rate. Abbreviations: AF, atrial fibrillation; ES, effect size; BVP, biventricular pacing; RVP, right ventricular pacing; CI, confidence interval.

The pooled AF incidence rate in other pacing strategies was 15.5% (95% CI: 9.6%–22.4%) [23, 24, 25, 26, 32]. Among all eligible studies, two reported the AF incidence rate in the BVP group (AF rate: 6.1%, 95% CI: 1.0%–14.0%) [25, 26], and three reported the AF incidence rate in the RVP group (AF rate: 19.4%, 95% CI: 14.1%–25.3%) (Fig. 3A) [23, 24, 32]. Long-term AF incidence rate in other pacing strategies was 15.5% (95% CI, 9.6%–22.4%), and short-term AF incidence rate was 5.9% (95% CI, 0.2%–15.9%) (Fig. 3B).

### 3.3 Risk of AF between LBBP and Other Pacing Strategies

**Fig. 4.**
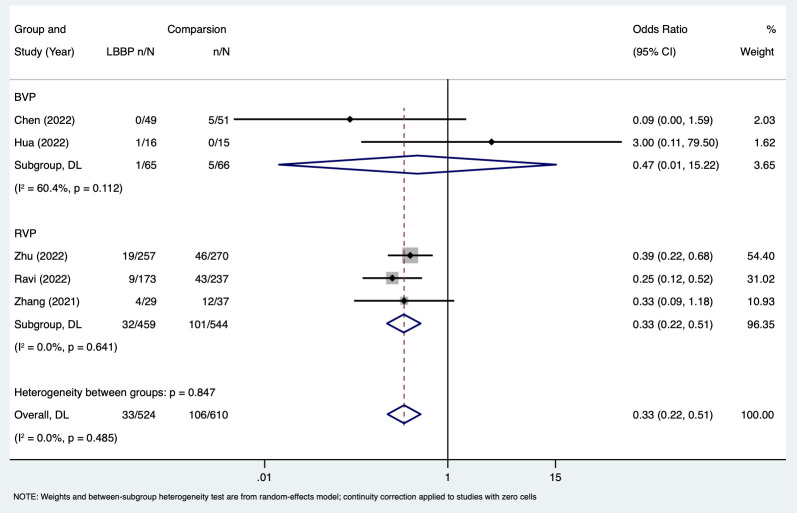
**Risk of AF in LBBP compared with other pacing strategies**. Abbreviations: AF, atrial fibrillation; LBBP, left bundle branch pacing; BVP, biventricular pacing; RVP, right ventricular pacing; CI, confidence interval; DL, DerSimonian-Laird.

**Fig. 5.**
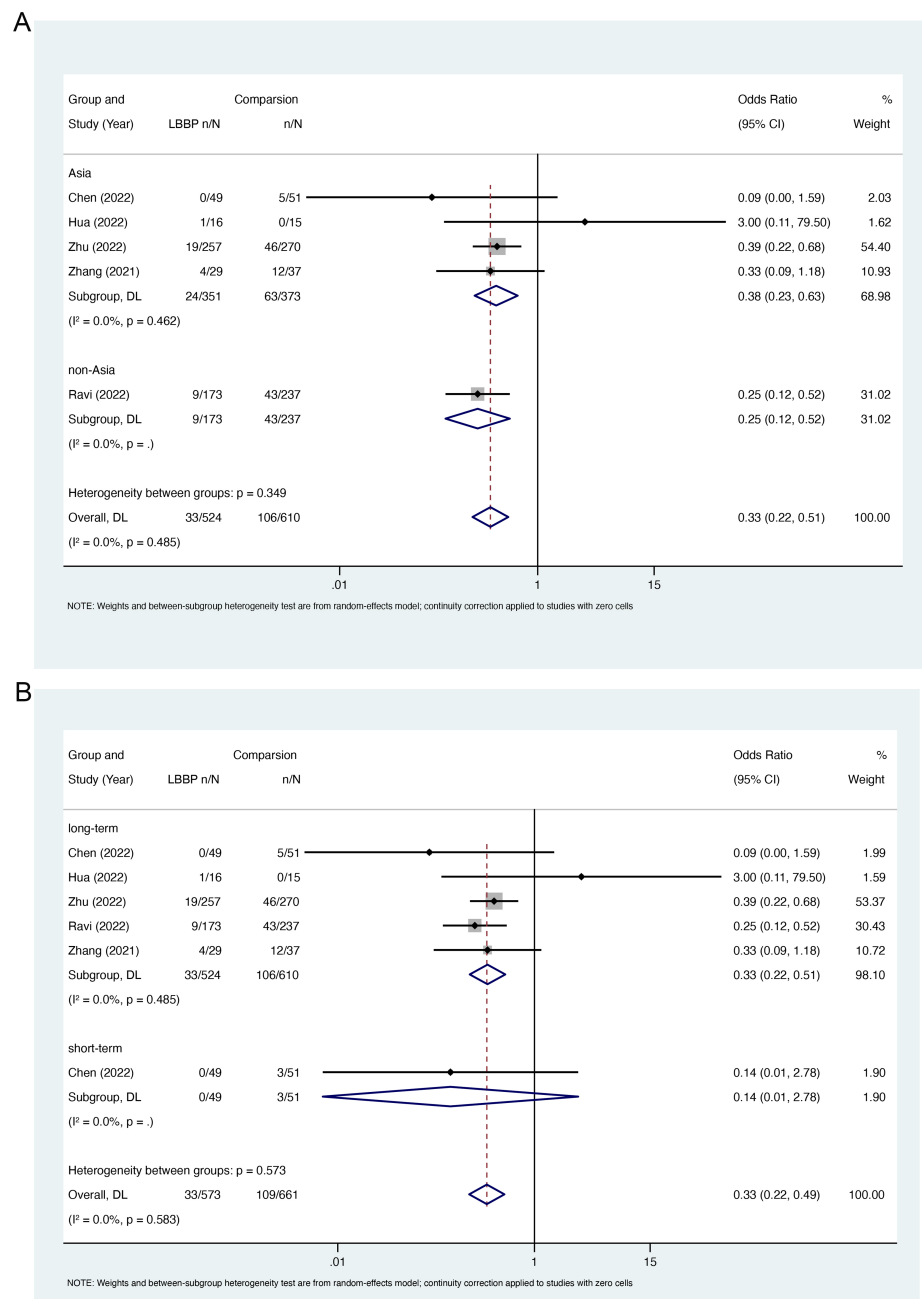
**Subgroup analysis of risk of AF in LBBP compared with other pacing strategies**. Subgroup analysis based on (A) race, (B) follow-up duration. Abbreviations: LBBP, left bundle branch pacing; DL, DerSimonian-Laird; CI, confidence interval.

For the comparison of the risk of AF between LBBP and other pacing strategies, the meta-analysis showed that LBBP was associated with a reduced risk of AF compared to other pacing strategies (OR, 0.33; 95% CI, 0.22–0.51; $I^{2}$ = 0.0%; *p* = 0.485) (Fig. 4). Similar benefits were observed for LBBP compared to BVP and RVP (Fig. 4). In addition, subgroup analyses showed that there was a reduced risk of AF following LBBP compared with that associated with other pacing strategies in Asian and non-Asian participants (Fig. 5A). For long-term AF risk, LBBP was associated with 67% risk reduction compared with other pacing strategies (OR, 0.33; 95% CI: 0.22–0.51, $I^{2}$ = 0.0%; *p* = 0.485). Similar benefits were observed with LBBP for short-term AF risk (Fig. 5B). In addition, two studies also reported the risk of AF between LBBP and RVP stratified by percentage of ventricular pacing (VP%) [23, 24]. Both studies suggested that the benefit of LBBP in reducing AF risk was more pronounced in patients with VP ≥20%.

**Fig. 6.**
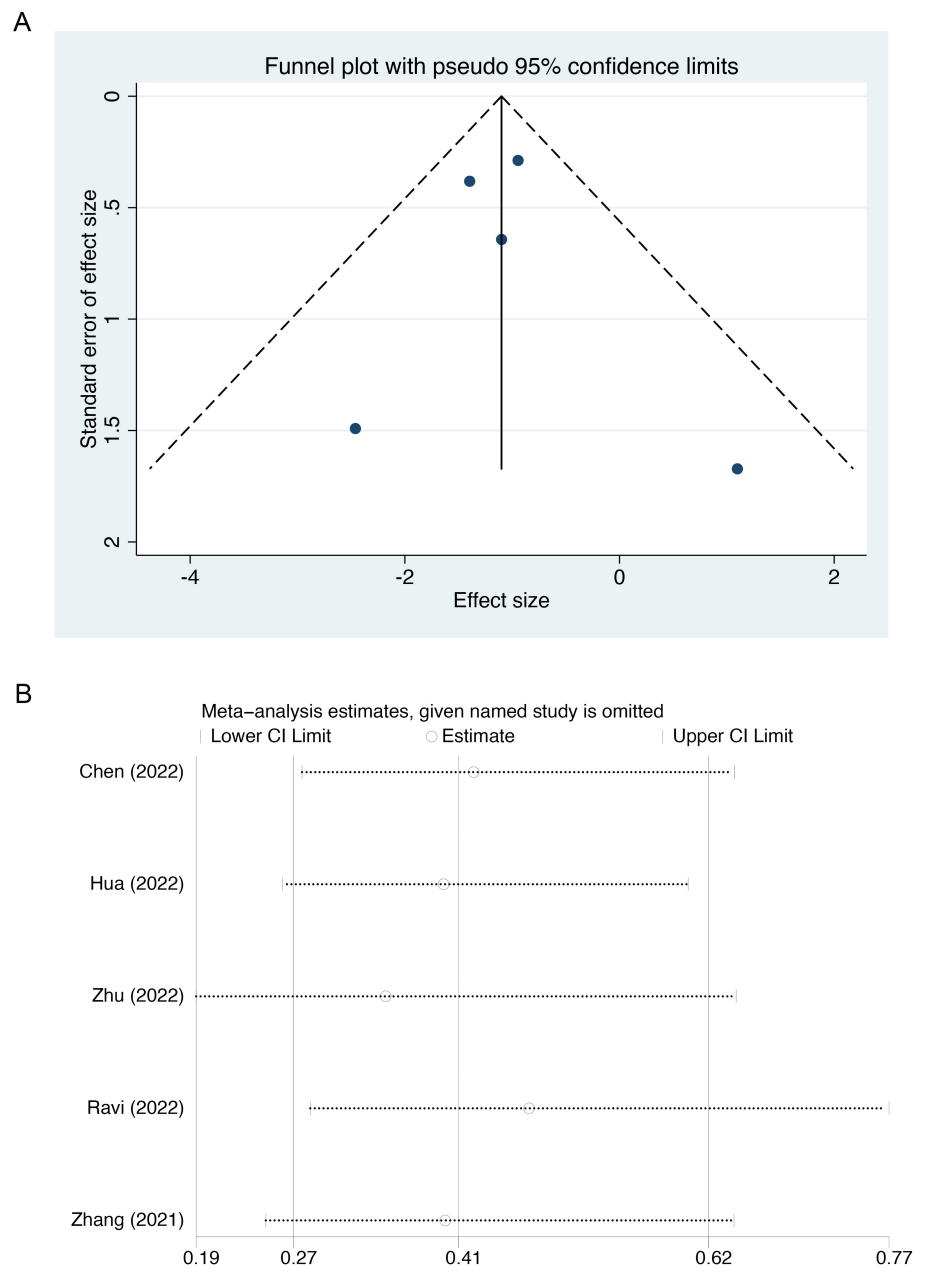
**Publication bias and sensitive analysis**. (A) Funnel plot. (B) Sensitive analysis.

The funnel plot was asymmetrical on both sides, indicating a lack of publication bias (Fig. 6A). Sensitivity analysis showed that there was no change in the combined results after excluding one study at a time (Fig. 6B).

## 4. Discussion

This systematic review and meta-analysis aimed to evaluate AF risk in LBBP compared with that in other pacing strategies. We found that the incidence of AF in the LBBP group was much lower than that in other pacing strategies (BVP and RVP). Compared to BVP or RVP, LBBP was associated with reduced AF risk. Similar outcomes of LBBP as regard AF have been observed among patients of different races. Our findings suggest that the risk of AF may be reduced in patients with LBBP compared with those with BVP or RVP.

It is well known that RVP is associated with an increased risk of AF. Although the exact mechanism of AF induced by ventricular pacing is not clear, it is generally accepted that left atrial dysfunction due to left ventricular dyssynchrony caused by ventricular pacing may be the cause [34]. BVP can maintain atrioventricular synchrony and better preserve physiological function, thereby reducing the risk of AF. The Mode Selection Trial in Sinus-Node Dysfunction (MOST) trial, which included 1014 sinus-node dysfunction (SND) patients with a median of 33.1 months of follow-up, found that dual-chamber pacing was associated with a 21% AF risk reduction (21.4% vs 27.1%; HR: 0.79 (0.66–0.94); *p* = 0.008) compared with ventricular pacing [35]. A subgroup analysis of the MOST trial involving patients with SND and normal QRS duration at baseline found a positive relationship between the cumulative percentage of VP (Cum%VP) and AF risk in both the dual-chamber pacing and ventricular pacing groups, which suggested that ventricular desynchronization induced by right ventricular apical pacing in the dual-chamber mode offsets the benefit of AV synchrony and increases AF risk [2]. The Search AV Extension and Managed Ventricular Pacing for Promoting Atrioventricular Conduction (SAVE PACe) trial further illustrated that dual-chamber minimal ventricular pacing was associated with an absolute risk reduction of 4.8% and a relative risk reduction of 40% for persistent AF compared with conventional dual-chamber pacing, indicating that reduced ventricular pacing in the dual-chamber mode prevents ventricular desynchronization [36]. Therefore, a more physiological pacing approach may help ameliorate ventricular dysfunction or atrioventricular asynchrony caused by ventricular pacing.

HBP maintains physiological electrical activation and reduces the risk of AF. Pastore *et al*. [37] illustrated that the location of RVP may affect the risk of AF, and the risk of AF in the Hisian area was lower than that in the right ventricular septal and apex (16.9% vs 25.7% vs 28.0%). Ravi *et al*. [24] found that HBP, compared with conventional RVP, was associated with a lower risk of new-onset AF, and the benefit was more pronounced in patients with a higher burden of ventricular pacing. However, significant difference in AF disease progression was observed between HBP and RVP in patients with previously diagnosed AF [38]. In addition, another study by Pastore *et al*. [39] further demonstrated that HBP was associated with a lower risk of persistent AF than a dual-chamber pacemaker with unnecessary ventricular pacing (DDD-VPA) and that the benefit of HBP was mainly found in patients with a basal PR greater than 180 ms. All these studies suggest that a more physiological pacing helps preserve AV dyssynchrony and thus prevent the onset of AF.

Therefore, LBBP, which belongs to the same physiological pacing category as HBP, can theoretically reduce the incidence of AF. Our findings indicate that LBBP is associated with a 67% reduction in AF risk compared with RVP, and a 53% reduction in AF risk compared with BVP. However, there are some limitations of this study. First, as LBBP is a relatively novel pacing technology, only 5 studies were included in our meta-analysis, of which 3 were prospective, 2 were retrospective, and none were RCTs, the small number of studies may yield biased results, especially for the AF incidence rate of BVP and RVP. Several large RCTs have reported the AF incidence involving BVP and RVP, which suggested that the AF incidence following BVP ranged from 8.5% to 23.0% [2, 35, 40, 41] and following RVP ranged from 9.3% to 28.0% [2, 35, 37]. Our studies estimated the AF incidence rate is 6.1% and 19.4% in BVP and RVP, respectively, and the incidence of AF in BVP may be underestimated. Data from real-world studies with larger samples are needed. Additionally, as none RCTs has reported the AF risk between LBBP and other pacing stratigies, large RCTs are needed to confirm the benefit of LBBP in reducing the risk of AF. Second, because the risk of AF varies and the effect of pacing varies in patients with different characteristics, more subgroup analyses according to patient characteristics are warranted. However, because individual data were not available, we only conducted subgroup analyses by Asian and non-Asian, long-term AF and short-term AF. Other subgroup analyses, such as with and without HF at baseline, need to be further explored. Furthermore, two studies showed that LBBP is more likely to be beneficial than RVP in patients with a higher ventricular pacing burden [23, 24]. Since QRS duration and PR intervals have been shown to be associated with an increased risk of AF [42, 43], further studies should also be conducted to determine whether the benefit of LBBP compared with BVP in reducing the risk of AF differs in patients with long versus short QRS duration, as well as long versus short PR intervals. Additionally, there is no comparative study of AF risk between LBBP and BVP under brady indication, which requires more research.

## 5. Conclusions

This meta-analysis found that LBBP, compared with BVP and RVP, was associated with a lower risk of AF. Whether LBBP is superior to other pacing strategies in reducing the risk of new-onset AF needs to be confirmed in large-sample randomized controlled trials.

## Data Availability

The original contributions presented in the study are included in the article/Supplementary Materials, further inquiries can be directed to the corresponding author.

## Supplementary Material

Supplementary material associated with this article can be found, in the online version, at https://doi.org/10.31083/j.rcm2408220.
